# A tuberculosis school outbreak in China, 2018: reaching an often overlooked adolescent population

**DOI:** 10.1017/S0950268819001882

**Published:** 2019-11-18

**Authors:** N. N. You, L. M. Zhu, G. L. Li, L. Martinez, W. Lu, Q. Liu, H. T. Yang

**Affiliations:** 1 Department of Epidemiology and Biostatistics, School of Public Health, Southeast University, Nanjing, Jiangsu Province, PR China; 2 Department of Chronic Communicable Disease, Center for Disease Control and Prevention of Jiangsu Province, Nanjing, Jiangsu Province, PR China; 3 Stanford University, School of Medicine, Division of Infectious Diseases and Geographic Medicine, Stanford, California, USA

**Keywords:** Adolescents, high school, homology, tuberculosis outbreak

## Abstract

Adolescents have been largely neglected from tuberculosis control efforts. In low- to medium burden settings much of the tuberculosis burden in this age group occurs from school outbreaks. We report on a large tuberculosis outbreak in adolescents from a boarding high school in Jiangsu Province, China. From March to June 2018, a tuberculosis outbreak occurred in a boarding high school. We conducted an outbreak investigation involving clinical diagnostic tests and molecular analysis to determine the outbreak origin. Cases were detected through symptom screening, tuberculin skin testing (TST), chest radiography, sputum smear, solid sputum culture and GeneXpert MTB/RIF. Mycobacterial interspersed repetitive-unit-variable-number tandem-repeat (MIRU-VNTR) genotyping and spoligotyping methods were performed on *Mycobacterium tuberculosis* (*M. tuberculosis*) isolates to identify the outbreak origin. A total of 845 students and 131 teachers/staff attended a TST screening for tuberculosis infection. The prevalence of elevated tuberculin reactions at ≥5, ≥10 and ≥15 mm was 12.19% (119/976), 6.35% (62/976) and 3.28% (32/976), respectively. Radiographic abnormalities were present in 5.73% (56 of 976) individuals, 40 students and 16 teachers/staff. Of these, 12 students were diagnosed with confirmed tuberculosis. In total, 14 students (two index cases and 12 confirmed cases) were diagnosed and reported in the tuberculosis outbreak, an attack rate of 1.7% (14/847) among students (two index cases and 845 screened students). Results from MIRU-VNTR typing and spoligotyping analyses demonstrated that three *M. tuberculosis* strains belong to the Beijing family with corresponding MIRU-VNTR alleles. This school-based tuberculosis outbreak among adolescents demonstrates that transmission among individuals in this age group is common and must be prioritised. It suggests that identifying and timely diagnosis of smear-positive cases, especially in the early phase of outbreaks, is the key to preventing further spread among close contacts.

## Introduction

Tuberculosis is a major cause of morbidity and mortality worldwide [1]. Historically, adults have been largely prioritised and heavily studied in the tuberculosis literature. Recently, more attention and research has focused on young children <10 years of age. However, adolescents between 10–20 years of age have been largely neglected. Tuberculosis case detection is likely low for this group compared to others [2, 3]. A recent report estimated that the global tuberculosis burden among adolescents was 1.78 million, accounting for almost 20% of global tuberculosis cases [4]. The tuberculosis literature is devoid of information on the risk of tuberculosis infection and disease progression among individuals in adolescence and this has impeded targeted control efforts [5].

In China, tuberculosis outbreaks often occur in institutional settings such as kindergartens, primary schools, high school and colleges [6, 7]. Compared to other school settings, boarding schools are subject to a higher risk of tuberculosis outbreaks, where adolescents often cluster under relatively overcrowded conditions [8, 9]. In developed countries with a low-tuberculosis burden, clustered tuberculosis outbreaks often occur in schools [10, 11]. In China, a large amount of work has been carried out on tuberculosis prevention and control in schools in recent years. Because laboratory diagnostic approaches have low yield in young children and teenagers, the diagnosis of tuberculosis in students primarily relies on clinical manifestations [6]. However, there have been few reports from China describing school-based tuberculosis outbreaks among adolescents [6, 12–14].

To inform this knowledge gap and investigate tuberculosis among adolescents, we describe a large tuberculosis outbreak in a boarding high school in Jiangsu Province, eastern China. We used a plethora of commonly used diagnostic tests as well as genotyping methods to inform recommendations for school tuberculosis outbreak control among adolescents in Chinese high schools.

## Methods

### Outbreak inception

Before the epidemic was confirmed, the Center for Disease Control and Prevention (CDC) staff responsible for epidemic monitoring found two cases of tuberculosis reported in the same high school on March 27. Subsequently, the local CDC staff went to the school to investigate the details of the two students with tuberculosis. Through the tuberculosis reporting system to query and track, then call the school to learn about the recent school absence among students found that these two cases are 12th grade students of the high school. The first case of tuberculosis, a 17-year-old female student with smear-negative tuberculosis, was reported on March 22nd. After 5 days, another 17-year-old female student was reported with smear-positive tuberculosis by her teacher who subsequently reported it to the relevant responsible agency. After this, an epidemiological investigation of tuberculosis cases and screening of contacts were initiated in the high school immediately.

### Study definitions

All cases were diagnosed by a respiratory, infectious disease clinician according to the *Chinese Guidelines for Tuberculosis Prevention and Treatment* [12]. Briefly, cases were classified into bacteriologically diagnosed, clinically diagnosed and tuberculosis suspects. Bacteriologically diagnosed cases were defined as either a positive smear, culture or pathological diagnosis of pulmonary lesions as tuberculosis. Anyone who met one of the following criteria was diagnosed as a clinically diagnosed case: smear-negative, a chest radiographic examination demonstrating pathological changes consistent with active tuberculosis accompanied by cough, expectoration, hemoptysis and other suspected symptoms of tuberculosis; smear-negative, chest radiographic examination showing pathological changes consistent with active tuberculosis, or a tuberculin skin test (TST) ≥15 mm, or vesicular necrosis, lymphatic inflammation; smear-negative, chest radiographic examination showing pathological changes consistent with active tuberculosis, or anti-tuberculosis antibody positive; smear-negative, chest radiographic examination showing pathological changes consistent with active tuberculosis, or extrapulmonary histopathological examination confirmed as tuberculosis; or a smear-negative tuberculosis suspect where other lung diseases after diagnostic treatment or follow-up observation. Anyone who met one of the following criteria could be diagnosed as a suspected case: only chest radiological appearances consistent with active tuberculosis; or children (age <5 years old) who have suspected symptoms of tuberculosis, accompanied by a history of close contact with smear-positive pulmonary tuberculosis or a TST induration result ≥15 mm. *M. tuberculosis* strains were typed and homologous analysed. We defined the strain with the same Mycobacterial interspersed repetitive unit variable number tandem repeat (MIRU-VNTR) alleles considered they had homology.

### Field investigation

After the initial two index cases, the CDC staff immediately conducted field epidemiological investigations and close contact screening. Close contacts with a TST above or equal to 15 mm were further investigated with a chest radiograph examination and were told to take prophylaxis if without disease. For those subjects without such a strongly positive TST took an additional TST after 3 months to detect individuals with tuberculosis infection who were still in the window period during the initial screening.

Systematic tuberculosis screening of contacts was conducted. A unified case questionnaire was used to case survey investigation, and data analysis was conducted by epidemiologists at the local CDC. Contacts were screened through clinical evaluations, TST and chest radiographs. If a chest radiograph was inconclusive, a computed tomography scan of suspicious lesions was obtained by experienced physicians. TST was performed by the county CDC staff. An intensive, close contact investigation was conducted on family members of confirmed patients, including asking about tuberculosis-related symptoms, TST and chest X-ray examinations. Individuals with an abnormal chest examination or suspicious symptoms were recommended for further testing with sputum smear or tuberculosis rapid diagnoses. Briefly, a trained doctor injected intradermally 0.1 ml (2 IU) of purified protein derivative (PPD) (Chengdu Biological Products Research Co., Ltd., Chengdu, China) into the inner surface of the left forearm. An experienced physician measured the transverse induration at the TST site 48–72 h after injection [15, 16]. TST results were categorised into ≥5, ≥10 and ≥15 mm induration reactions. Chest radiographs were performed on all the students, and teachers/staff in the school at the local county people's hospital. Among those with imaging abnormalities suspicious of tuberculosis, sputum smears and cultures were further collected and subjected to laboratory testing.

Acid-fast staining by the Ziehl–Neelsen method and cultures using Lowenstein–Jensen media. In addition, GeneXpert MTB/RIF (Xpert, Cepheid, USA) were performed on all collected sputum specimens, which is recommended by the World Health Organization as the initial test in children, adolescents and people living with HIV [17]. Briefly, 1 ml of sputum sample was mixed with 2 ml GeneXpert MTB/RIF sample reagent and incubated at room temperature for 15 min. Next, 2 ml of mixture was added to a test cartridge and loaded onto the GeneXpert MTB/RIF instrument following the manufacturer's instructions [12].

### MIRU-VNTR and spoligotyping

*M. tuberculosis* isolates collected from bacteriological diagnosed cases were performed by MIRU-VNTR typing methods as previously reported [18], the number of repeats at each locus is recorded and reported in the order MIRUs 2, 4, 10, 16, 20, 23, 24, 26, 27, 31, 39 and 40, MTUBs 04, MTUBs 21 as well as QUB11b. Briefly, the polymerase chain reaction (PCR) fragments were analysed by 1.5% agarose gel electrophoresis with a 100-bp DNA ladder, and the copy number at each locus was calculated using the Quantity One gel imaging system. Determination of the discriminatory power of the VNTR locus was calculated using the Hunter–Gaston discriminatory index. A standard strain of H37Rv was used as a positive control.

Spoligotyping of isolates was performed as described by Kamerbeek *et al*. [19]. In brief, genomic DNA of *M. tuberculosis* isolates was amplified using the PCR with the primers of Dra (biotin labelled) and Drb, and then the PCR products were hybridised to a set of 43 oligonucleotide probes corresponding to each spacer, which were covalently bound to a membrane. A standard strain of H37Rv was used as a positive control.

### Statistical analysis

Characteristics included age, sex, sputum smear, culture and GeneXpert MTB/RIF tests. TST results were processed at 5, 10 and 15 mm cut-off values. All statistical analysis was conducted using SPSS software (version 19.0, International Business Machines Corporation, IBM). The data were collected and extracted by Categorical data were compared by the *χ*^2^ test or Fisher's exact test as appropriate. *P*-Values < 0.05 were considered statistically significant.

### Ethics statement

This project was approved by the Institutional Review Board of Jiangsu Provincial Center for Disease Control and Prevention. Written informed consent was obtained from all participants.

## Results

### Population setting

**Fig. 1.**
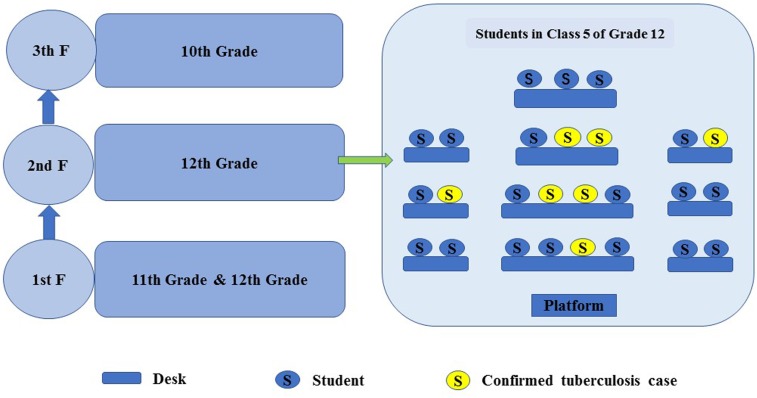
Distribution map of students.

The school is a provincial senior high school with 847 students. The high school incorporates three grades: 186 students in the 10th grade with four classes, 249 students in the 11th grade with six classes and 412 students in the 12th grade with ten classes. These students' age ranged between 15 and 20 years old; the mean age was 17.73 (standard deviation, ± 1.21) years old. In this school, there are 140 teachers/staff and a school clinic with one school doctor. The tuberculosis outbreak occurred mainly in the 12th grade, which is the last year of high school, facing great pressure of college entrance examination. There are 309 resident students and 91 dormitories; male and female students live in two separate dormitory buildings with an average of four persons per room. The female dormitory building is located in the right rear of the teaching building, sharing corridors and stairs. The 12th grade female students live mainly on the second floor while female students from other grades live on other floors. The male student dormitory is located in the northeast corner of the school, sharing corridors and stairs. The 12th grade male students live predominantly on the second floor (Fig. 1). The canteen area, where students have meals together, is about 400 square meters and well ventilated.

### Index case finding

In March 2018, a 17-year-old female student was reported with tuberculosis. She was in Class 5 of the 12th grade of the high school. She attended a local clinic because of hemoptysis on March 8, she was given anti-inflammatory treatment and, after one week, after which her symptoms did not improve. On March 15, she was considered a tuberculosis suspect in the county's Second People's Hospital. She ultimately was identified as a smear-negative tuberculosis patient on March 22, and anti-tuberculosis treatment was subsequently carried out DOTS at home.

On March 23, another 17-year-old female student from the high school attended the Second People's Hospital for coughing and was diagnosed as a suspected tuberculosis patient. Then she was diagnosed with new sputum-smear positive pulmonary TB and hospitalised in the tuberculosis designated hospital on March 27. She resided in Class 2 of the 12th grade and she also carried out DOTS at home.

### Contact investigation

After confirmation of these two tuberculosis cases, local health authorities conducted an initial contact investigation of the same classes of the index cases from this high school. A total of 81 close contacts (74 students and seven teachers) of the index cases were evaluated using TST and chest radiographs from March 27 to April 9. Seven tuberculosis suspects among 47 students were identified in Class 5 of the 12th grade, among them six were confirmed as tuberculosis cases. Meanwhile, one student with chest abnormalities was confirmed as a smear-negative tuberculosis patient among 27 students in Class 2 of the 12th grade on April 17.

After being confirmed of these additional cases, it already met the definition of cluster epidemic (≥3 tuberculosis cases), so the scope of contact investigation was extended covering the entire floor of the classes and dormitories where the index cases occurred. From April 12 to April 20, 595 individuals (496 students and 99 teachers/staff) were additionally screened, among whom additional five tuberculosis suspects were confirmed as tuberculosis with laboratory examination results and clinical characteristics.

**Fig. 2.**
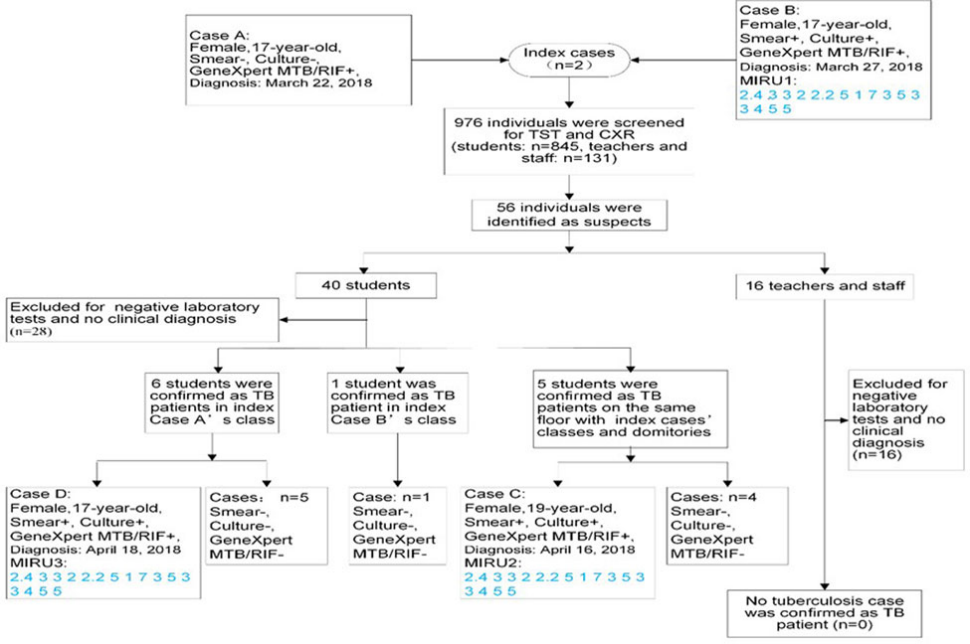
Diagnosis and exclusion of all suspected cases found during the whole investigation of close contacts in the school. Smear−, smear-negative; Smear+, smear-positive; Culture−, culture-negative; Culture+, culture-positive; GeneXpert MTB/RIF−, GeneXpert MTB/RIF negative; GeneXpert MTB/RIF+, GeneXpert MTB/RIF positive; TST, tuberculin skin testing; CXR, chest radiography/chest X-ray; TB, tuberculosis.

**Fig. 3.**
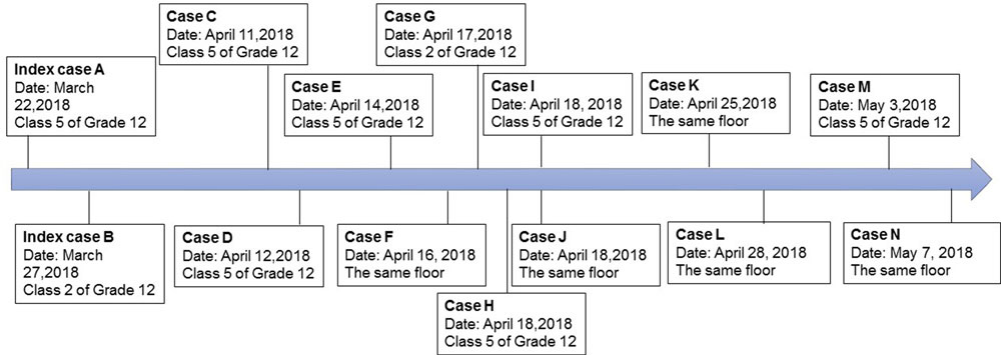
Timeline of confirmed tuberculosis cases in a high school in Jiangsu Province, China.

Through these two contacts screening, 12 tuberculosis cases were identified among students, it was considered that there were preliminary assessment elements constitute public health emergency and should be promptly disposed according to relevant regulations, and the scope of contact investigation was extended covering the entire school. From April 21 to April 28, screening was again extended to cover the entire school. An additional 300 individuals (275 students and 25 teachers/staff) were screened, of which 13 were identified as tuberculosis suspects, however no tuberculosis patient identified after clinical diagnosis (Figs 1–3). During the close contact investigation, no tuberculosis patients were identified among family members of confirmed patients.

In total, 976 individuals were screened as contacts from March 27 to April 28. The positive rate of TST ≥5 mm was 12.19% (119/976), TST ≥10 mm was 6.35% (62/976) and TST ≥15 mm was 3.28% (32/976) (Table 1). A total of 14 pulmonary tuberculosis cases (including two index cases) were reported among students. Of these, three cases were smear-positive and 11 cases were smear-negative. GeneXpert MTB/RIF was performed on 56 suspected patients and two index cases, of which four were positive and others were negative, of which three cases were positive of sputum culture (Table 2). All cases were first treated with the standardised first-line regimen (2HRZE/4HR) in the local tuberculosis hospital. Preventive therapy was recommended for those with no evidence of active tuberculosis but with a TST ≥15 mm [20]. In addition, all patients completed treatment or were cured and no secondary patients subsequently occurred.

**Table 1.** Screening of close contacts between teachers and students in the high school

Group	Total No.		Tuberculin skin test results				Chest X-ray/CT Abnormal results	Suspected cases	Confirmed cases
			<5 mm	≥5 mm	≥10 mm	≥15 mm			
Student	845		779	44	25	40	40	40	12
Teachers/staff	131		78	75	37	16	16	16	0
Total		976	857	119	62	56	56	56	12

CT, computed tomography.

Suspected cases: only chest radiological appearances consistent with active tuberculosis; or children (age <5 years old) who have suspected symptoms of tuberculosis, accompanied by a history of close contact with smear-positive pulmonary tuberculosis or a TST induration result ≥15 mm.

**Table 2.** Characteristics of the tuberculosis suspects and confirmed cases in the outbreak, Jiangsu, China

Characteristic	Tuberculosis suspects and two index cases (No.)		Tuberculosis (No.)	Healthy (No.)
	Students	Teachers/staff		
Sex				
Female	25	6	9	22
Male	17	10	5	22
Age (mean)				
Students	17.61 ± 1.41	/	17.93 ± 1.00	17.37 ± 1.64
Teachers/staff	/	47.00 ± 8.60	/	47.00 ± 8.60
Sputum smear status				
Positive	3	0	3	0
Negative	39	16	11	44
Sputum culture				
Positive	3	0	3	0
Negative	39	16	11	44
GeneXpert MTB/RIF				
Positive	4	0	4	0
Negative	38	16	10	44
Total	42	16	14	44

### Laboratory results

Fifty-six specimens among suspected cases and two index cases in this epidemic were collected and performed sputum smear in county people's hospital, of which three were sputum smear positive. Meanwhile, all sputum samples were submitted to the Jiangsu provincial CDC for GeneXpert MTB/RIF and sputum culture testing. Of these sputum specimens, four cases were positive for GeneXpert MTB/RIF detection and three cases were positive for culture (one GeneXpert MTB/RIF positive case was culture negative due to the low level of positivity). MIRU-VNTR and spoligotyping results demonstrated that all these three *M. tuberculosis* strains belonged to the Beijing family and contained the same MIRU-VNTR alleles (Appendix Table A1).

### Ethical approval

This study was reviewed and approved by the Ethics Committee of Jiangsu Province Center for Disease Control and Prevention. Written informed consent was obtained from all study participants.

### Analysis of the epidemic

There were 14 confirmed cases among students (nine female, five male), and the attack rate was 1.65% (14/847) among students. Of them, 13 cases were concentrated in the 12th grade and one case in the 11th grade in the high school, the attack rate of the 12th grade students was 3.16% (13/412), the attack rate of the first index case’s class was 25% (7/28), the attack rate of second index case's class was 4.17% (2/48), the overall attack rate of the second index cases' classes was 11.84% (9/76), which was 11.15 times higher than that of other classes' students in the 12th grade (*χ*^2^ = 19.659, *P* < 0.001) (Table 3). According to the result of MIRU-VNTR, three *M. tuberculosis* were confirmed to be infected by the same strain, and there was a molecular epidemiological relationship between these cases. Therefore, this school tuberculosis cluster epidemic was a homologous outbreak in accordance with the results of field epidemiology and molecular epidemiology.

**Table 3.** Tuberculosis incidence of students in the high school

Location of student	*N*	Confirmed cases			Attack rate No.（%）
		No. of cases	Male	Female	
Class 5 of Grade 12	28	7	3	4	25
Class 2 of Grade 12	48	2	1	1	4.17
Other classes of Grade 12	336	4	1	3	1.19
Grade 11	249	1	0	1	0.40
All students	847	14	5	9	1.65

Class 5 of Grade 12, Class 5 of the 12th grade; Class 2 of Grade 12, Class 2 of the 12th grade; Other classes of Grade 12, Other classes of the 12th grade; Grade 11, the 11th grade.

## Discussion

By conducting a detailed epidemiological investigation and molecular genotyping, we described a school tuberculosis outbreak in a county of southeast China. Most pre-college students have great academic pressure due to the nationwide college entrance examinations and the lack of physical exercise in China. Numerous previous relevant literatures reported crowded living, insufficient ventilation, persistent contact with tuberculosis cases and diagnostic delay as the primary factors causing pulmonary tuberculosis outbreaks among high school students [11, 21, 22]. Our results showed that students who resided in the same class as the index case were at a significantly higher risk than other students. Therefore, the priority concern is given to the students who were in the same class as the index case. In addition, female students were found to have a higher risk to progress active tuberculosis compared to male students possibly due to the fact that two index cases were female.

MIRU-VNTR has a high utilisation value for the molecular epidemiology classification of *M. tuberculosis* with known epidemiological association, and it shows superior discrimination ability [23].There are many studies on the molecular epidemiological investigation of MIRU-VNTR for tuberculosis [18, 24, 25]. In the study of Black *et al*. the MIRU-VNTR genotyping method can be used to track and determine whether a homologous outbreak has occurred between cases [24]. Norheim *et al*. conducted a study on tuberculosis outbreaks in an educational institution in Norway [10]. They performed MIRU-VNTR genotyping on the strains of the confirmed patients, and 22 of the 24 patients had the same VNTR site, confirming that it was a homologous outbreak. In the study of Lu Wei *et al*. genotyped the strains based on a new 15-locus MIRU-VNTR method in combination with spoligotyping technology demonstrated a high discriminatory power [18]. It is useful for the epidemiological analysis of MTB transmission. However, we should also note that field epidemiology is still the key to track the epidemic. Although MIRU-VNTR strain typing was vital in identifying linked cases, epidemiological information was essential in understanding disease transmission. The enhanced cluster questionnaire and interviewing skills of TB nurses identified possible transmission settings.

This was the first study to use MIRU-VNTR and spoligotyping genotyping methods to investigate the source case in a school tuberculosis outbreak in our province. In this outbreak, we used a spoligotyping method combined with the new MIRU-15 technique, and the results of the obtained three strains of MIRU-VNTR genotyping showed that the 15 loci of the three strains were completely identical, indicated that these three patients were part of the same outbreak, consistent with the above findings. Although we only got three *M. tuberculosis* isolates in this outbreak, however the six secondary confirmed cases were in the same class as the first index case, which was epidemiologically related in location, time and spatial distribution. Moreover, the result of MIRU-VNTR indicated that the three *M. tuberculosis* isolates were a homologous source. Although the three patients were in different classes, there was an intersection between the self-study of many classes and eat in the canteen together.

The serious diagnostic delay of the first index case of tuberculosis maybe was the main reason of this outbreak. This delay partly was due to the clinical manifestations of tuberculosis which are easily confused with symptoms of respiratory tract infections that often resolve after antibiotic treatment [26]. Before the first case was diagnosed as tuberculosis, she had appeared upper respiratory symptoms, a frequent cough and fever in early February 2018. Between February 20 and March 8, as the illness was aggravated, she saw a doctor in a small local clinic on March 8. After a long period of anti-inflammatory treatment and no improvement of symptoms on March 15, she was diagnosed as suspected tuberculosis then referred to the county tuberculosis designated Hospital. She was confirmed as a patient with smear-negative pulmonary tuberculosis on March 22. The period from the onset of tuberculosis symptoms to the diagnosis of tuberculosis in the first index case lasted about two months. Previous studies have shown that the diagnostic delay of the first case is the main cause of the tuberculosis outbreak [11, 12], which is also fully demonstrated that the incidence of students in the same class as the first case is significantly higher than that of other classes in this epidemic. The distance between contact and index patients is an important driver of *M. tuberculosis* transmission. As shown in the seat map of the first patient's class, most of student cases were seated adjacent to the index case. Therefore, we believe that the probability of long-term exposure to index cases put certain students at high risk of infection and subsequent development of tuberculosis. During this period, the first index case occasional took leave to see a doctor, but also continued to attend classes and stay in school, which greatly increased the risk of tuberculosis transmission [24]. School health supervision measures such as morning check-up, registration of absenteeism due to illness and tracking system have not been effectively implemented.

We conducted a close contact survey of family members of the two index cases and found no tuberculosis patients or individuals with a previous history of tuberculosis. Therefore, we speculate that the first patient may have been infected with *M. tuberculosis* in a community setting. The class of the second index case was adjacent to the first index patient's class – therefore, it's possible transmission occurred when they participated in public self-study classes, in canteen, or campus activities. In addition, the time from the symptom onset to tuberculosis diagnosis lasted two months for the first index case. Teachers and students have less knowledge about tuberculosis and no preventive measures were taken during this time.

China is a country with universal bacille Calmette–Guerin (BCG) vaccination. Accompanied by 14 tuberculosis cases reported, the attack rate of the first index case to members of her class was 25%. Despite this, the rate of TST results ≥15 mm was not as high as expected (3.28% in the school), which did not play a better role in the early warning for the outbreak. The strong positive rate of TST is not high may be due to the following reasons. Firstly, the detection ability of PPD may vary depending on the production produced by different pharmaceutical companies or different batches produced by the same company. Secondly, nurses are not trained systematically for TST, and there may be irregularities in usage; thirdly, though BCG is the main immune mean to prevent and reduce the occurrence of tuberculosis, its immune effect also has deficiencies and lastly, the skin reaction of PPD has a window period, generally 12 weeks [27, 28], and rapidly initiated TST for close contacts may lead to negative reactions in some infected individuals. A high degree of *M. tuberculosis* exposure is essential for transmission and secondary patients [29], and foreign studies have also shown that the incidence of tuberculosis is high in close contacts, whether TST are positive or not [30].

The sputum specimen of the first index case was positive for GeneXpert MTB/RIF detection, despite the fact that *M. tuberculosis* was not cultured and isolated. The reason may be that the patient had carried out anti-tuberculosis treatment for a period of time when the sputum specimens were sent to the provincial CDC. *M. tuberculosis* may have been killed resulting in no strain growth during culture.

A few limitations of this outbreak investigation are worth mentioning. First, we failed to culture the *M. tuberculosis* strain of the first index case to perform molecular typing methods. Second, we did not perform genome-wide sequencing analysis, which limited our understanding of the transmission network and likely affected the inference about transmissibility [6].

In summary, this school-based tuberculosis outbreak among adolescents demonstrates that transmission among individuals in this age group is common and must be prioritised. First, we should pay more attention to health education in schools and raise awareness of teachers and students about tuberculosis. Once chronic cough occurs, teachers should consider the possibility of tuberculosis. Second, chest radiograph examinations should be incorporated into school freshmen entrance medical examination. Faculty and staff must do chest radiograph examinations every year. Third, illness absence monitoring and cause tracking of students to achieve early detection of tuberculosis case. Fourth, local CDC should monitor school tuberculosis cases as an early warning system. Once tuberculosis cases occur in school, further close contact screening and follow-up work can be performed. Early diagnosis is paramount. The problem is identifying and timely diagnosis of smear-positive cases, especially in the early phase of the outbreak, is the key to preventing further spread among close contacts.

## Appendix

**Table A1.** MIRU-VNTR genotyping results of 15 locus of three *M. tuberculosis* isolates.

Categories	MIRU2	MIRU4	MIRU10	MIRU16	MIRU20	MIRU23	MIRU24	MIRU26	MIRU27	MIRU31	MIRU39	MIRU40	MTUB04	MTUB21	QUB11b
Case B	2.4	3	3	2	2.2	5	1	7	3	5	3	3	4	5	5
Case C	2.4	3	3	2	2.2	5	1	7	3	5	3	3	4	5	5
Case D	2.4	3	3	2	2.2	5	1	7	3	5	3	3	4	5	5
H37Rv	2.4	3	3	2	2.2	5	1	7	3	5	3	3	4	5	5

Case B, a 17-year-old female student in Class 2 of the 12th grade is also an index case; Case C, a 19-year-old female student in Class 8 of the 12th grade, the same floor with the index cases; Case D, a 17-year-old female student in Class 5 of the 12th grade, the same class of the first index case; H37Rv, a standard strain.
